# Retinoic acid receptor alpha mediates growth inhibition by retinoids in rat pancreatic carcinoma DSL-6A/C1 cells.

**DOI:** 10.1038/bjc.1998.673

**Published:** 1998-11

**Authors:** F. H. Brembeck, A. Kaiser, K. Detjen, H. Hotz, T. Foitzik, H. J. Buhr, E. O. Riecken, S. Rosewicz

**Affiliations:** Department of Gastroenterology, Medizinische Klinik I, Klinikum Benjamin Franklin, FU Berlin/Germany.

## Abstract

**Images:**


British Journal of Cancer (1998) 78(10). 1288–1295
© 1998 Cancer Research Campaign

### Retinoic acid receptor α mediates growth inhibition by retinoids in rat pancreatic carcinoma DSL-6A/C1 cells

**FH Brembeck[1], A Kaiser[1], K Detjen[1], H Hotz[2], T Foitzik[2], HJ Buhr[2], E-O Riecken[1] and S Rosewicz[1]**

Department of Gastroenterology. [1]Medizinische Klinik I. [2]Department of Surgery. Klinikum Benjamin Franklin. FU Berlin/Germany. Hindenburgdamm 30. 12200 Berlin. Germany

**Summary** During carcinogenesis, pancreatic acinar cells can dedifferentiate into ductal adenocarcinoma of the pancreas. DSL-6A/C1 cells represent an in vitro model of this carcinogenic sequence. This study was designed to examine the effects of retinoids on cell growth in DSL-6A/C1 cells and to characterize further the molecular mechanisms underlying the antiproliferative actions of retinoids. Treatment of DSL-6A/C1 cells with retinoids results in a time- and dose-dependent inhibition of cell growth, paralleled by a retinoid-mediated transactivation of a pTK::βRAREx2-luciferase reporter construct transiently transfected into DSL-6A/C1 cells. Retinoid receptor expression was evaluated by reverse transcriptase polymerase chain reaction (RT-PCR) using subtype-specific primers and demonstrated expression of retinoic acid receptor alpha (RAR-α), RAR-β and retinoid X receptor alpha (RXR-α). Using a panel of receptor subtype-specific agonists, the RAR-α specific agonist Ro 40-6055 was the most potent retinoid in terms of growth inhibition. Furthermore, all-*trans*-retinoic acid-mediated growth inhibition and transactivation was completely blocked by the RAR-α-specific antagonist Ro 41-5253. In summary, the RAR-α subtype predominantly mediates the antiproliferative effects of retinoids in DSL-6A/C1 cells. Furthermore, this cell system provides a feasible tool to study the molecular mechanisms underlying the growth inhibitory effects of retinoids in ductal pancreatic carcinoma cells derived from a primary acinar cell phenotype.

**Keywords:** ductal pancreatic carcinoma; all-*trans*-retinoic acid; retinoic acid receptor α

Despite intensive clinical trials during the last decades, therapy of advanced human exocrine pancreatic carcinoma remains unsatisfactory: only few chemo- and radiochemotherapeutic regimen have produced modest survival benefits in patients with advanced disease (Lionetto et al. 1995). Therefore, alternative treatment strategies have focused on substances which exert their anti-tumorigenic effects by growth inhibition and induction of cellular differentiation, rather than cytotoxicity. In this context, retinoids have recently emerged as a novel therapeutic strategy for the treatment of human pancreatic carcinoma (Rosewicz et al. 1995*a*; Brembeck et al. 1998). Previous studies were performed using cultured human ductal pancreatic carcinoma cell lines, because more than 90% of human pancreatic cancers are histologically classified as ductal adenocarcinoma of the exocrine pancreas. However, the pathogenesis of pancreatic cancer remains unsolved, especially regarding the cell type of origin (acinar versus duct cell) as the primary progenitor of the carcinogenic pathway. A carcinogen-induced model in the Syrian golden hamster results in primary ductal pancreatic carcinoma, harbouring a point mutation at codon 12 of the K-*ras* gene, as is characteristically observed in the majority of human tumours (Cerny et al. 1990); other animal models of pancreatic carcinoma suggest that this malignancy is probably not derived from a primary duct cell origin because it could be demonstrated that acinar cells upon malignant transformation can dedifferentiate to a duct-like cell type in some rodents (rats and mice) without aquisition of K-*ras* point mutations (Dissin et al. 1975; Longnecker and Curphey, 1975; Bockman et al. 1976, 1978; Satake et al. 1984; Scarpelli et al. 1984; De Lisle and Logsdon, 1990; Longnecker et al. 1992; Pettengill et al. 1993). As an in vitro model for this 'transdifferentiation' theory of pancreatic carcinogenesis, Pettengill et al (1993) established the DSL-6A/C1 cell line from an azaserine-induced acinar carcinoma of the rat and obtained a cell line with duct cell-specific properties by regrafting the tumour cells from in vivo to in vitro and vice versa. Although not experimentally proven, it appears conceivable that this carcinogenic sequence might also apply for human pancreatic carcinoma given that more than 90% of the human exocrine pancreas is represented by acinar cells. Therefore, the aim of our study was to establish this cell line as a suitable in vitro model to investigate the molecular mechanisms involved in retinoid-mediated growth inhibition.

Retinoids, which are summarized as natural and synthetic derivatives of vitamin A, have been demonstrated to inhibit proliferation and induce differentiation in a variety of malignant tissues (Lotan, 1980; Bollag, 1983; Lotan et al. 1990; Love and Gudas, 1994). Clinical trials demonstrated beneficial effects of retinoids in the treatment of various premalignant lesions and malignancies (reviewed by Bollag and Holdener, 1992; Smith et al. 1992; Tallman and Wiernik, 1992). The molecular action of retinoids was partially elucidated within the past decade. After passive diffusion through the cell membrane, retinoids interact with two cytoplasmic retinoid-binding proteins, which are believed to regulate intracellular retinoid homeostasis (Giguère, 1994). Retinoids

*Received 4 November 1997*
*Revised 5 February 1998*
*Accepted 16 April 1998*

*Correspondence to:* S Rosewicz. Universitätsklinikum Benjamin Franklin der Freien Universität Berlin. Medizinische Klinik I (Gastroenterologie/Infektiologie). Hindenburgdamm 30. D-12200 Berlin. Germany exert their pleiotropic effects on cell growth and differentiation via two families of nuclear retinoid receptors: the retinoic acid receptors (RAR) and the retinoid X receptors (RXR) (Umesono, 1991; Giguère, 1994). Each retinoid receptor family consists of three different subtypes (α, β, γ) encoded by distinct genes (Mangelsdorf et al, 1994). The RAR subtypes can further be divided in multiple isoforms because of gene transcription via different use of two internal promoter sites or, alternatively, by different splicing of the mRNA transcripts (Kastner et al, 1990; Zelent et al, 1991; Giguère, 1994). The distinct receptor subtypes are expressed in a cell type-specific pattern during embryogenesis, in the adult organism as well as in malignant tissues. It has therefore been concluded that retinoid sensitivity of a given tissue is defined in part through the expression pattern of RAR/RXR subtypes (Giguère, 1994).

RARs are bound and activated by the naturally occurring retinoid all-*trans*-retinoic acid (Leid et al, 1992*a*; Giguère, 1994). In contrast, RXRs are predominantly activated by 9-*cis*-retinoic acid (Heyman et al, 1992; Leid et al, 1992*b*), whereas ATRA does not bind to these receptors (Mangelsdorf et al, 1992). Binding of 9-*cis*-RA is necessary to form RXR homodimers or heterodimers of the RXRs with the RARs.

Retinoid receptors are part of the nuclear vitamin D–thyroid hormone receptor superfamily, which act as ligand-activated transcription factors of retinoid-regulated genes and gene networks (Green and Chambon, 1988). The ligand-activated RARs and RXRs bind as dimers to their responsive elements, the retinoic acid responsive elements (RAREs) or retinoid X responsive elements (RXREs) (Leid et al, 1992*a*). Activation or suppression of retinoid-regulated genes is mainly mediated through the binding of RAR/RXR heterodimers to a 'retinoic acid responsive element' (RARE) within the promoter region of such genes (Näär et al, 1991; Umesono et al, 1991; Durand et al, 1992; Kliewer et al, 1992). Only RAR/RXR heterodimers have been shown to bind effectively to a 'retinoic acid responsive element' of retinoid-regulated genes (Leid et al, 1992*a*). In addition, retinoid receptors interact with a variety of co-activators and co-repressors, thereby either relieving repression by the basal transcription machinery or inducing ligand-dependent transcription above the basal level (Mangelsdorf and Chambon, 1995).

Therefore, in a given cell or tissue, retinoid sensitivity as well as the spectrum of biological effects elicited by retinoids are determined by the expression pattern of the nuclear retinoid receptor subtypes and by different heterodimeric RAR/RXR combinations. Consequently, for the understanding of retinoid-mediated growth inhibition in pancreatic carcinoma cells, it appears necessary to characterize the retinoid receptor subtype expression as well as the biological function of individual retinoid receptor subtypes. In the present study, we therefore evaluated the molecular effects of retinoids in the cell line DSL6A/C1 as a model for ductal pancreatic carcinoma derived from primary acinar cells. Furthermore, we identified the particular receptor subtype responsible for retinoid-mediated control of proliferation in this pancreatic carcinoma cell line.

### MATERIALS AND METHODS

### Materials

The rat pancreatic carcinoma cell lines DSL-6A/C1 and AR42J were obtained from the American Type Tissue Culture Collection (ATCC, Rockville, USA) and the human pancreatic carcinoma cell line Dan-G from Deutsches Krebsforschungszentrum (DKFZ, Heidelberg, Germany); Dulbecco's modified Eagle medium (DMEM) was obtained from Gibco BRL (Berlin, Germany) and fetal calf serum (FCS) from Biochrom (Berlin, Germany). [$\alpha^{32}$P]dCTP (6000 Ci mmol$^{-1}$) was purchased from DuPont (Bad Homburg, Germany); Random priming labelling kit and HybondN+ membranes were obtained from Amersham (Braunschweig, Germany). RNA molecular size markers, random hexamer primers, Moloney murine leukaemiavirus reverse transcriptase (M-MLV), T4 polynucleotide kinase and restriction enzymes were obtained from Bethesda Research Laboratories (BRL, Bethesda, USA). *Thermus aquaticus (Taq)* DNA polymerase was purchased from Promega (Heidelberg, Germany). The cDNA probes for the human RAR-α, RAR-β, RAR-$\gamma_1$, RXR-α, RXR-β and mouse RXR-γ were kindly provided by P Chambon (Strasbourg, France); the pTK::βRAREx2-luc construct was kindly provided by R Evans (San Diego, USA); all retinoids were kindly provided by Hofmann-LaRoche (Basle, Switzerland). All other chemicals were of analytical grade and purchased from Sigma (Deisenhofen, Germany).

### Cell culture

Cells were grown under 95% air and 5% carbon dioxide at 37°C as subconfluent monolayers in DMEM or RPMI-1640 supplemented with either 20% v/v (AR42J) or 10% v/v (Dan-G and DSL-6A/C1) FCS, penicillin (100 U ml$^{-1}$) and streptomycin (100 μg ml$^{-1}$). All experiments were carried out in the log phase of growth after the cells were seeded for 24 h. Preparation of retinoids as stock solutions was performed under subdued light and aliquots were kept at –80°C for a maximum of 4 weeks. DMSO was used as a solvent for all retinoids. Control cells were treated with the same amount of DMSO and the final concentration of DMSO in the culture medium did not exceed 0.1% (v/v) Cell viability in growth assays was routinely checked by trypan blue staining and was routinely found to be >95%.

### RNA isolation and Northern blot analysis

Total RNA was isolated using the RNAzol reagent (WAK Chemie Medical, Bad Homburg, Germany) according to the instructions of the manufacturer. Qualitative analysis of RNA was performed using Northern blot analysis as previously described (Rosewicz et al, 1994). RNA was denatured in a buffer containing 6% v/v formaldehyde and 5% v/v formamide, subjected to electrophoretic separation in a 1% agarose gel containing formaldehyde and then transferred to a nylon membrane. A RNA ladder was electrophoresed in parallel for size determination. The cDNA probes for synaptophysin (Rosewicz et al, 1992*a*), human carbonic anhydrase II (Rosewicz et al, 1995*b*) and amylase (Rosewicz et al, 1989) have been previously described. The cDNA inserts were eluted by glasmilk with the Geneclean II kit (Dianova, Hamburg, Germany). cDNA probes were radioactively labelled with [$\alpha^{32}$P]dCTP using the random primer labelling kit following the instructions provided by the supplier. Unincorporated nucleotides were removed by passing the reaction sample through a Sephadex G50 (LKB Pharmacia, Bromma, Sweden) column. The specific activity of the probes was usually $1\text{–}2 \times 10^9$ c.p.m. μg$^{-1}$ DNA. Hybridization of membranes was performed using the Quickhyb reagent (Stratagene, Heidelberg, Germany) according to the manufacturer's instructions. After hybridization, membranes were sequentially washed with increasing stringency with 2 × SSC and 1 × SSC (1 × standard saline citrate = 0.15 M sodium chloride, 0.015 M sodium citrate, pH 7.0) with 0.1% sodium dodecyl sulphate (SDS) at room temperature, and finally with 0.1 × SSC/0.1% SDS at 65°C for 15 min each wash. Membranes were then exposed to radiographic film for 1–3 days using two intensifying screens.

### Anchorage-dependent growth assay

Single cell suspensions of trypsinated cells were plated in 96-well culture dishes at a density of 3000 cells per well in the presence of supplemented culture medium. After an attachment time of 12 h, retinoids were added from stock solutions at the indicated concentrations. Control cells were treated with vehicle alone. At the indicated time points, cells were gently washed twice with phosphate buffered saline (PBS, Gibco BRL, Berlin, Germany) and then harvested by trypsinization. Viable cells were counted in a haemocytometer by trypan blue exclusion. Triplicate wells were analysed for each data point.

### Reverse transcriptase polymerase chain reaction (RT-PCR) analysis

Reverse transcription of RNA from DSL-6A/C1 cells was performed using 1 μg total RNA, 100 pM random hexamer primer, 1 mM dithiothreitol, 6 mM $Mg^{2+}$, 500 μM each of dNTP, 20 U RNAsin (Promega, Heidelberg, Germany), and murine leukaemia virus reverse transcriptase. This reaction mixture was promptly used as template for the PCR at a 1:20 dilution. For amplification, the receptor subtype specific primers complementary to human RAR-α, RAR-β, RAR-γ, RXR-α, RXR-β and mouse RXR-γ nucleotide sequences were exactly used as previously described (Rosewicz et al, 1995*a*). The reaction was carried out in 10 mM Tris-HCl buffer (pH 9.0), containing 50 mM potassium chloride, 0.01% Triton X-100, 1 mM magnesium chloride (1.5 mM $MgCl_2$ for RAR-α), 200 μM each of dNTP, 50 pM of each primer and 2.5 U *Taq* DNA polymerase in a final volume of 50 μl. Thirty-five cycles of amplification were carried out as follows: denaturation 30 s at 92°C, annealing 90 s at 60°C, extension 90 s at 72°C and final extension at 72°C for 10 min. As an internal negative control, one RNA aliquot was amplified without prior reverse transcription to ensure that the amplified PCR product was not due to amplification of contaminating genomic DNA.

### Transient transfections and transactivation assays

DSL-6A/C1 cells were transiently transfected with the pTK::βRAREx2-luc reporter construct. This bluescript plasmid contains the luciferase reporter gene (Forman et al, 1995) under the control of the herpesvirus thymidine kinase promoter containing two tandem copies of the retinoic acid responsive element (RARE) of the human RAR-β promoter which is followed by the SV40 polyadenylation signal. For transient transfection of DSL-6A/C1 cells, 300 000 cells were plated in six-well culture dishes. After an attachment period of 12 h, cells were incubated with 1 ml of the transfection mixture. This mixture contained 10 μg plasmid DNA and 100 μl Lipofectamine reagents (Gibco BRL, Eggenstein, Germany) in a total volume of 18 ml serum-free DMEM medium. Cells were incubated for 6 h, followed by change of the culture medium with DMEM containing 10% FCS and the retinoids at the indicated concentrations. Control cells transfected

**Figure 1** Expression of cell type-specific markers in pancreatic carcinoma cells (Northern blot analysis). The expression of the ductal cell marker carbonic anhydrase II, the acinar cell marker amylase and synaptophysin as a neuroendocrine-specific marker were characterized in the human ductal pancreatic carcinoma cell line Dan-G, the rat amphicrine AR42J cells and the rat DSL-6A/C1 tumour cell line. Total RNA (30 μg) was isolated and analysed by Northern blotting as described in Materials and methods. Ethidium bromide stains of the gels before and after transfer to the membrane were obtained, confirming that equal amounts of RNA for each cell line were hybridized. Shown is a representative of three independent experiments yielding identical results. The molecular size indicated on the right side was deduced from a RNA ladder electrophoresed in parallel

**Figure 2** Time-dependent effects of all-*trans*-retinoic acid on cell growth in DSL-6A/C1 cells. Proliferation of DSL-6A/C1 cells after stimulation with 10 μM ATRA was determined by cell counts in an anchorage-dependent growth assay over a period of 4 days. Means (±s.e.m.) of absolute cell number of triplicates in three independent experiments are indicated for vehicle-treated control cells and ATRA-stimulated cells. *Significant differences at the $P < 0.05$ level with the same reporter plasmid were incubated with vehicle alone. Twenty-four hours later, cell lysates were obtained and luciferase activity of lysates was determined using the luciferase reagent (Promega, Heidelberg, Germany) by measurement of 'relative luciferase units' (RLU) in a luminometer. Results are expressed as x-fold induction over basal activity of vehicle treated controls.

 © Cancer Research Campaign 1998

**Figure 3** Dose-dependent effects of all-*trans*-retinoic acid on cell growth (**A**) and transactivation of a RARE reporter construct (**B**). (**A**) Anchorage-dependent growth assay: DSL-6A/C1 cells were incubated with the indicated concentrations of ATRA over 96 h. Cell counts were determined and expressed as per cent of vehicle-treated control cells. Shown are the means (± s.e.m.) of three independent experiments, each performed in triplicates. **P*-values <0.05 vs control. (**B**) Transactivation assays: DSL-6A/C1 cells were transiently transfected with the pTK::βRAREχ2-luc reporter construct and stimulated with the indicated concentrations of ATRA. After a total of 48 h from the beginning of transfection, luciferase activity of transfected cells stimulated with vehicle or ATRA was determined by measurement of 'relative luciferase units' in a luminometer. Luciferase activity is expressed as x-fold induction (means ± s.e.m.) over basal activity of vehicle-treated controls ($n = 3$; $P < 0.05$)

### Statistical analysis

Statistics were performed by the unpaired, one-sided Student's *t*-test. Differences were considered statistically different at the $P < 0.05$ level. All data are shown as means ± s.e.m.

### RESULTS

### DSL-6A/C1 express duct cell-specific markers

To verify the ductal phenotype of DSL-6A/C1 cells, we initially examined the expression of cell type-specific markers in two well-characterized pancreatic carcinoma cell lines, AR42J and Dan-G, and compared them with DSL-6A/C1 cells. We have previously described the AR42J cell line as an amphicrine pancreatic carcinoma cell line, which combines exocrine acinar with neuro-endocrine features (Rosewicz et al, 1992*b*). The ductal phenotype of Dan-G cells has previously been confirmed by cytokeratin phenotyping (Rosewicz et al, 1995*a*). Using a cDNA for the pancreatic acinar cell-specific human amylase in Northern blot analysis, we detected a single hybridization band with a molecular size of 1.8 kb only in AR42J cells, but not in Dan-G or in DSL-6A/C1 cells (Figure 1). Hybridization with a cDNA probe for the neuro-endocrine vesicle membrane-specific protein synaptophysin reveals a hybridization signal of approximately 2.8 kb only in AR42J cells, but could not be detected in Dan-G or in DSL-6A/C1 cells (Figure 1). Using a human cDNA probe for carbonic anhydrase II (CAII), which is specifically expressed in pancreatic duct cells, we obtained a hybridization signal of about 2.0 kb both in Dan-G cells and in DSL-6A/C1 cells, but not in AR42J cells (Figure 1). Therefore, DSL-6A/C1 cells, although derived from a pancreatic acinar carcinoma, fail to express pancreatic acinar and neuroendocrine markers, but do express CAII – a marker gene specifically restricted to duct cells of the pancreas (Kim et al, 1990).

### Effects of all-*trans*-retinoic acid on cell growth and transactivation of a RARE reporter construct in DSL-6A/C1 cells

As an initial attempt to determine the responsiveness of a dedifferentiated pancreatic acinar cell line to the antiproliferative effects of retinoids, we examined the effects of the naturally occurring retinoid all-*trans*-retinoic acid (ATRA) on anchorage-dependent growth in DSL-6A/C1 cells. We incubated DSL-6A/C1 cells with ATRA over a period of 4 days and determined cell number every 24 h. For initial experiments, we chose 10 μM ATRA because this represents the concentration which can be achieved after oral uptake of this substance at maximal, non-toxic plasma levels in humans (Muindi et al, 1992). After incubation with ATRA, we observed a significant time-dependent growth inhibition in DSL-6A/C1 cells as early as 2 days (74.9±7.4% of control, $n = 3$, $P < 0.05$; Figure 2). Furthermore, the antiproliferative effects of ATRA are dose dependent, with half-maximal growth inhibition observed at 100 nM ATRA (77.5±10.8% of control, $n = 6$, $P < 0.05$) and maximal inhibition at 10 μM ATRA (55.7 ± 7.6% of control, $n = 6$, $P < 0,05$; Figure 3A).

Having shown that DSL-6A/C1 cells are sensitive in terms of ATRA-mediated growth inhibition, we next decided to evaluate the transcriptional activation of retinoid-regulated genes in this cell line to gain further evidence as to whether retinoid-regulated gene transcription accounts for the antiproliferative effects of ATRA. We therefore investigated the effects of ATRA on transactivation of a retinoic acid responsive element linked to a luciferase reporter gene. We transiently transfected DSL-6A/C1 cells with the pTK::βRAREx2-luciferase reporter construct. Stimulation of transient transfected DSL-6A/C1 cells with ATRA resulted in a dose-dependent stimulation of luciferase activity with a maximum at 10 μM ATRA (78.6±9.9% over control, $n = 3$, $P < 0.05$; Figure 3B). These data demonstrate that incubation of DSL-6A/C1 cells

© Cancer Research Campaign 1998

**Figure 4** Dose-dependent effects of retinoid analogues on proliferation of DSL-6A/C1 cells. Proliferation of DSL-6A/C1 cells was determined by cell counts after incubation with the respective retinoid at the indicated concentrations over 120 h. All data are expressed as per cent of vehicle-treated control cells. Shown are the means (± s.e.m.) of three independent experiments, each performed in triplicates $^{*}P < 0.05$ vs control

**Figure 5** Expression of nuclear retinoid receptors in DSL-6A/C1 cells (RT-PCR analysis). Using receptor subtype specific oligonucleotide primers, expression of retinoid receptors (RAR/RXR) in DSL-6A/C1 cells was characterized by RT-PCR. Size determination as indicated on the left was performed using a 100-bp DNA ladder (M). As a negative internal control, one RNA aliquot was amplified without prior reverse transcription (–); the respective complementary DNA for each receptor subtype (cDNA) served as a positive control. Shown is a representative of 3–5 independent RT-PCR reactions with ATRA results in growth inhibition and transactivation at comparable concentrations of ATRA, suggesting a tight correlation between retinoid-regulated gene expression and the ATRA-induced growth inhibition in DSL-6A/C1 cells.

### Effects of retinoid analogues on proliferation of DSL-6A/C1 cells

We next asked which nuclear retinoid receptor family might be responsible for the ATRA-mediated growth inhibition and transcriptional control of retinoid responsive genes in DSL-6A/C1 cells. We therefore compared the effects of the retinoid analogues ATRA, 13-*cis*- and 9-*cis*-RA on anchorage-dependent growth. Figure 4 demonstrates the dose-dependent retinoid effects on anchorage-dependent growth of DSL-6A/C1 cells. Maximal antiproliferative effects could be observed with 10 μM of each retinoid analogue. The biological activity of these retinoids on an equimolar basis (at 10 μM 120 $h^{-1}$) in DSL-6A/C1 is as follows: ATRA > 13-*cis*-RA > 9-*cis*-RA. At lower concentrations (<1 μM), only 13-*cis*-RA and ATRA exerted similar growth inhibitory effects, whereas 9-*cis*-RA failed to demonstrate a significant growth inhibition (Figure 4). Although isomerization and degradation have to be considered, these observations suggest that retinoid-mediated growth inhibition in DSL-6A/C1 cells might be due to activation of the RAR receptor family rather than the RXR pathway.

### Expression of nuclear retinoid receptors in DSL-6A/C1 cells

We next examined the expression pattern of nuclear retinoid receptors to gain more information about which retinoid receptor subtype might mediate retinoid action in this cell line. To characterize the RAR/RXR subtype expression pattern, we applied the very sensitive RT-PCR analysis using receptor subtype-specific primers because retinoid receptors are often expressed as low abundance mRNAs, thereby barely detectable by standard Northern blot analysis. As an internal positive control RT-PCR was performed with the respective receptor cDNA yielding amplification products of the expected size for each receptor subtype (Figure 5). The validity of the primers for rat tissues has previously been described (Rosewicz et al, 1995*a*).

To exclude amplification signals arising from contaminating genomic DNA, each PCR was initially performed without prior reverse transcriptase reaction and repeatedly found to be negative. Using the receptor subtype-specific primers we detected expression of RAR-α, RAR-β and RXR-α, whereas no amplification signal for RAR-γ, RXR-β and RXR-γ could be detected (Figure 5).

### Effects of RAR-specific agonists on proliferation of DSL-6A/C1 cells

Data obtained from anchorage-dependent growth assays using different retinoid analogues indicate that only retinoids, which predominantly activate the RARs, result in significant growth inhibition of DSL-6A/C1 cells at lower concentrations. We therefore assumed that ATRA-mediated effects on growth and transactivation are mediated by a subtype of the RAR family expressed in DSL-6A/C1 cells (RAR-α or RAR-β). We examined, therefore, the effects of RAR subtype-specific retinoids on anchorage-dependent growth of DSL-6A/C1 cells using the RAR-α subtype-specific

 © Cancer Research Campaign 1998

**Figure 6** Effects of RAR-specific agonists on growth in DSL-6A/C1 cells. Cell growth of DSL-6A/C1 cells was determined by cell counts after incubation with the RAR-α selective agonist Ro-40-6055, the RAR-β selective agonist Ro 48-2249 and ATRA. Cells were incubated with the respective retinoid at the indicated concentrations over 96 h. Means (± s.e.m.) of three independent experiments, each performed in triplicate, are expressed as per cent of vehicle-treated control cells. $^*P < 0.05$ vs control

**Figure 7** The RAR-α antagonist Ro 41-5253 blocks ATRA-mediated growth inhibition and transactivation of the pTK::βRAREχ2-luc reporter construct. (**A**) Anchorage-dependent growth assay: DSL-6A/C1 cells were incubated with the indicated concentrations of the RAR-α antagonist Ro 41-5253 and 100 nM ATRA over 96 h. Cell counts were determined and expressed as per cent of vehicle-treated control cells. Shown are the means ± s.e.m. of three independent experiments, each performed in triplicates. * *P*-values <0.05 vs ATRA-mediated growth inhibition. (**B**) Transactivation assays: DSL-6A/C1 cells were transiently transfected with the pTK::βRAREχ2-luc reporter construct and stimulated with 100 nM ATRA with or without the presence of the RAR-α antagonist Ro 41-5253. Luciferase activity of transfected cells ($n = 3$) was determined in a luminometer and expressed as x-fold induction (means ± s.e.m.) over basal activity of vehicle treated controls; $^*P$-values < 0.05 vs ATRA-mediated induction of luciferase activity retinoid Ro 40–6055 and the RAR-β-specific agonist Ro 48-2249. Both agonists have been shown to bind and activate preferentially the corresponding receptor subtype (Crettaz et al, 1990). The antiproliferative effects of the RAR-α-specific agonist were comparable to the growth-inhibitory effects of ATRA on an equimolar basis: 100 nM Ro 40-6055 (RAR-α specific agonist) 77.0±3.1% of control ($n = 3$, $P < 0.05$; Figure 6) versus 100 nM ATRA 76.2±3.3% of control ($n = 3$, $P < 0.05$; Figure 6). In contrast, the RAR-β-specific retinoid Ro 48-2249 was mainly ineffective.

### Effects of the RAR-α-specific antagonist Ro 41-5253 on ATRA-mediated growth inhibition and transactivation

To further substantiate the role of the RAR-α receptor subtype in mediating the effects of ATRA in DSL-6A/C1 cells, we used the RAR-α-specific antagonist Ro 41-5253 in growth and transactivation assays. This retinoid has been shown to revert, selectively, RAR-α-mediated retinoid effects (Apfel et al, 1992). Incubation of DSL-6A/Cl cells with this antagonist at the maximal concentration used in our experiments had no effect on growth (data not shown). However, ATRA-mediated growth inhibition can be blocked by the RAR-α-specific antagonist Ro 41-5253 in a dose-dependent manner, with a complete growth inhibition observed at an antagonist concentration of 1–10 µM (Figure 7A). Similar effects could also be observed when we investigated ATRA-mediated transactivation of the pTK::βRAREx2-luc reporter construct in transiently transfected DSL-6A/C1 cells: incubation with 10 µM RAR-α-specific antagonist Ro 41-5253 completely blocks the stimulation of luciferase activity induced by 100 nM ATRA (Figure 7 B).

### DISCUSSION

Retinoids have been shown to exert profound effects on cellular growth and differentiation in a variety of malignant cells in vitro and in vivo (Lotan, 1980; Bollag, 1983; Lotan et al, 1990). Moreover, retinoids have been proven clinically beneficial in certain premalignant lesions (e.g. dysplasias of the cervix, the respiratory tract, in the head and neck area) and in various malignancies (for review see Bollag and Holdener, 1992). We have previously shown that retinoids induce cellular differentiation and inhibit proliferation of human ductal carcinoma cells in vitro and in vivo (Rosewicz et al, 1995*a*). In addition, combination therapy of retinoic acid with interferon α in patients with advanced, unresectable carcinoma of the pancreas results in prolonged survival of responsive patients (Brembeck et al, 1998). However, the molecular basis of retinoid action in pancreatic carcinoma cells is still incompletely understood, especially with regard to which retinoid receptor subtype might account for the antiproliferative effects of retinoids.

To further dissect this problem, we therefore decided to investigate the molecular effects of retinoids in the pancreatic carcinoma cell line DSL-6A/C1, because this cell system provides the unique features of a primary acinar pancreatic carcinoma which has dedifferentiated into a ductal phenotype (Pettengill et al, 1993). Some experimental evidence suggests that, at least in some rodents, this sequence of malignant transformation might best reflect the in vivo situation of pancreatic carcinogenesis (Dissin et al, 1975; Longnecker and Curphey, 1975; Bockman et al, 1976, 1978; Satake et al, 1984; Scarpelli et al, 1984; De Lisle and Logsdon, 1990; Longnecker et al, 1992).

The ductal phenotype of DSL-6A/C1 cells was confirmed by characterizing the expression pattern of pancreatic cell type-specific marker genes compared with two other well-defined pancreatic carcinoma cell lines, Dan-G and AR42J. Dan-G cells represent a retinoid-sensitive, human ductal pancreatic carcinoma cell line (Rosewicz et al, 1995*a*). AR42J cells were established from an azaserine-treated rat model and represent a retinoid-insensitive, acinar pancreatic carcinoma cell line with neuroendocrine features (Rosewicz et al, 1992*b*, 1995*a*). By Northern blot

*© Cancer Research Campaign 1998*

analysis, we demonstrated expression of the acinar cell marker amylase and the neuroendocrine marker gene synaptophysin, whereas the ductal cell marker carbonic anhydrase II (CAII) is not expressed in the amphicrine cell line AR42J. In contrast, DSL-6A/C1 cells (despite their origin from an azaserine-induced acinar pancreatic carcinoma) have lost the expression of the acinar marker amylase, but express the duct cell-specific marker CAII. Expression of synaptophysin in these cells could not be detected, indicating a lack of transdifferentiation to a neuroendocrine phenotype. These results are in good agreement with the study of Pettengill et al (1993) regarding the histopathological features and the expression pattern of cytokeratins, indicating that DSL-6A/C1 cells represent a pancreatic carcinoma cell line with a bona fide ductal phenotype.

Having confirmed the ductal phenotype of DSL-6A/C1 cells, we next investigated whether these cells are retinoid sensitive in terms of growth inhibition. Using an anchorage-dependent growth assay, we observed that treatment with ATRA results in a time- and dose-dependent growth inhibition. Compared with 13-*cis*-RA and 9-*cis*-RA, ATRA was the most potent antiproliferative retinoid. Somewhat less pronounced growth inhibitory effects were observed for the stereoisomer of ATRA, 13-*cis*-RA, whereas the retinoid analogue 9-*cis*-RA failed to inhibit cell growth at lower, therapeutically relevant concentrations (<100 nM). These results indicate that retinoid-mediated growth inhibition is mainly due to activation of the RAR receptor family because ATRA predominantly activates the RAR subtypes (Heyman et al, 1992; Leid et al, 1992*a*, 1992*b*; Giguère, 1994).

We next focused on the molecular basis of ATRA-mediated effects in DSL-6A/C1 cells and tested the ability of ATRA to stimulate gene transcription of a synthetic RARE reporter construct. In transient transfection assays with the pTK::βRAREx2-luc construct, we demonstrated that ATRA dose-dependently mediates transactivation of this reporter construct. The dose–response curve of ATRA-mediated transactivation correlates tightly with the growth inhibitory effects observed in the anchorage-dependent growth assays. These data are compatible with the hypothesis that antiproliferative effects of retinoids are mediated by the transcriptional control of genes regulating cellular growth. However, it should be kept in mind that the effects of retinoids might also be mediated by transcriptional repression as well as functional interference with the transcription factor AP-1 (Giguere, 1994).

To achieve further insight into the molecular mechanism of retinoid action in this cell system, we characterized the expression of the RAR and RXR subtypes. To our surprise, we found a very restricted expression pattern of RAR/RXR subtypes in DSL-6A/C1 cells. Using the highly sensitive RT-PCR technique, we detected two RAR subtypes (RAR-α and RAR-β) and only one member of the RXR family (RXR-α). RAR-γ, RXR-β and RXR-γ are not expressed in these cells. The lack of RXR-γ expression is not surprising because this subtype exhibits a very restricted expression pattern in only a few tissues (Dolle et al, 1990), and is also not expressed in a broad panel of human pancreatic carcinoma cell lines (Rosewicz et al, 1995*a*). In contrast to the DSL-6A/C1 cells, all previously characterized human ductal pancreatic carcinoma cell lines express the RAR-γ as well as the RXR-β (Rosewicz et al, 1995*a*). The retinoid-insensitive rat amphicrine carcinoma cell line AR42J shows, similar to the retinoid-sensitive DSL-6A/C1 cells, no expression of the RAR-γ. The selective loss of the RAR-γ subtype might be due to the malignant transformation of acinar cells because this receptor subtype is expressed in normal pancreatic acinar cells (Xu et al, 1996).

Each receptor subtype might fulfill a distinct biological function in a given cell by activating a panel of responsive genes (Giguère, 1994). We therefore investigated which receptor subtype might be responsible for ATRA-mediated growth inhibition in DSL-6A/C1 cells. Because ATRA predominantly activates the RAR subtypes and DSL-6A/C1 cells only express RAR-α and RAR-β, we investigated the effects of two RAR subtype-specific retinoids on anchorage-dependent growth: Ro 40-6055, a specific agonist of the RAR-α subtype; and Ro 48-2249, a RAR-β specific agonist (Crettaz et al, 1990). Only the RAR-α specific agonist exerted dose-dependent antiproliferative effects similar to equimolar concentrations of ATRA, whereas the RAR-β-specific retinoid Ro 48-2249 had no antiproliferative effects at concentrations less than 1 μM. This suggests an important role for the RAR-α subtype in mediating the effects of ATRA in DSL-6A/C1 cells.

If the RAR-α subtype is responsible for growth inhibition and transactivation of the RARE reporter construct in DSL-6A/C1 cells, it should be possible to block the ATRA-mediated effects by antagonizing the RAR-α subtype. To test this hypothesis, we used the highly specific RAR-α antagonist Ro 41-5253 (Apfel et al, 1992) in anchorage-dependent growth and transactivation assays. ATRA-mediated growth inhibition as well as transactivation of the RARE reporter construct could be completely blocked by the RAR-α antagonist, suggesting that RAR-α is crucial for mediating the biological effects of ATRA in DSL-6A/C1 cells.

Recent studies indicated that the expression pattern of distinct retinoid receptor subtypes determines retinoid-mediated effects in a given tissue (Giguère, 1994). For example, RAR-α is found to be responsible for growth inhibition in breast cancer cells (van der Leede et al, 1995), whereas RAR-β mediates antiproliferative effects in fibroblasts (Lee et al, 1992) and RAR-γ is predominantly responsible for growth inhibition by retinoids in human teratocarcinoma (Moasser et al, 1994) or neuroblastoma (Marshall et al, 1995). Despite this divergence in different tissues, the identification of a distinct receptor subtype mediating the antiproliferative effects of retinoids in a given cell type provides a powerful therapeutic tool to potentiate the antiproliferative effects and minimize side-effects potentially mediated by other receptor subtypes. Based on the different retinoid receptor expression pattern between rat and human pancreatic carcinoma cells, the implications of this study for experimental therapy of human pancreatic cancer have to be interpreted cautiously. However, the results of the present study are indicative that receptor subtype-specific retinoids (i.e. RAR-α-specific agonists) might be an attractive alternative to all-*trans*-retinoic acid in the experimental treatment of pancreatic carcinoma.

In summary, we have demonstrated that the antiproliferative effects of retinoids in the ductal pancreatic carcinoma cell line DSL-6A/C1 are predominantly mediated by the RAR-α subtype. Moreover, DSL-6A/C1 cells as a model of ductal adenocarcinoma derived from dedifferentiated pancreatic acinar cells provide a feasible in vitro system to study the molecular mechanisms underlying growth inhibitory effects of retinoids in ductal pancreatic carcinoma cells.

### ACKNOWLEDGEMENT

This work was supported by a grant from the Deutsche Krebshilfe to Stefan Rosewicz (10-0954-Ro2).

 © Cancer Research Campaign 1998

### REFERENCES

Apfel C, Bauer F, Crettaz M, Forni L, Kamber M, Kaufmann F, LeMotte P, Pirson W and Klaus M (1992) A retinoic acid receptor α antagonist selectively counteracts retinoic acid effects. *Proc Natl Acad Sci USA* **89**: 7129–7133

Bockman DE, Black Jr O, Mills LR, Mainz DL and Webster PD (1976) Fine structure of pancreatic adenocarcinoma induced in rats by 7,12-dimethylbenz(a)anthracene. *J Natl Cancer Inst* **57**: 931–936

Bockman DE, Black Jr O, Mills LR and Webster PD (1978) Origin of tubular complexes developing during induction of pancreatic adenocarcinoma by 7,12-dimethylbenz(a)anthracene. *Am J Pathol* **90**: 645–658

Bollag W (1983) Vitamin A and retinoids: from nutrition to pharmacotherapy in dermatology and oncology. *Lancet* **1**: 860–863

Bollag W and Holdener EE (1992) Retinoids in cancer prevention and therapy. *Ann Oncol* **3**: 513–526

Brembeck FH, Schoppmeyer K, Leupold U, Gornistu C, Keim V, Mössner J, Riecken EO and Rosewicz S (1998) A phase II pilot trial of 13-cis retinoic acid and interferon-α in patients with advanced pancreatic carcinoma. (*submitted*)

Cerny WL, Mangold KA and Scarpelli DG (1990) Activation of K-ras in transplantable pancreatic ductal adenocarcinomas of Syrian golden hamsters. *Carcinogenesis* **11**: 2075–2079

Crettaz M, Baron A, Siegenthaler G and Hunziker W (1990) Ligand specificities of recombinant retinoic acid receptors RAR α and RAR β. *Biochem J* **272**: 391–397

De Lisle RC and Logsdon CD (1990) Pancreatic acinar cells in culture: expression of acinar and ductal antigens in a growth-related manner. *Eur J Cell Biol* **51**: 64–75

Dissin J, Mills LR, Mains DL, Black Jr O and Webster PD (1975) Experimental induction of pancreatic adenocarcinoma in rats. *J Natl Cancer Inst* **55**: 857–864

Dolle P, Ruberte E, Leroy P, Morriss-Kay G and Chambon P (1990) Retinoic acid receptors and cellular retinoid binding proteins. I. A systematic study of their differential pattern of transcription during mouse organogenesis. *Development* **110**: 1133–1151

Durand B, Saunders M, Leroy P, Leid M and Chambon P (1992) All-trans and 9-cis retinoic acid induction of CRABPII transcription is mediated by RAR–RXR heterodimers bound to DR1 and DR2 repeated motifs. *Cell* **71**: 73–85

Forman BM, Umesono K, Chen J and Evans RM (1995) Unique response pathways are established by allosteric interactions among nuclear hormone receptors. *Cell* **81**: 541–550

Giguère V (1994) Retinoic acid receptors and cellular retinoid binding proteins: complex interplay in retinoid signaling. *Endocrine Rev* **15**: 61–79

Green S and Chambon P (1988) Nuclear receptors enhance our understanding of transcription regulation. *Trends Biochem Sci* **4**: 309–314

Heyman RA, Mangelsdorf DJ, Dyck JA, Stein RB, Eichele G, Evans RM and Thaller C (1992) 9-cis retinoic acid is a high affinity ligand for the retinoid X receptor. *Cell* **68**: 397–406

Kastner P, Krust A, Mendelsohn C, Garnier JM, Zelent A, Leroy P, Staub A and Chambon P (1990) Murine isoforms of retinoic acid receptor γ with specific patterns of expression. *Proc Natl Acad Sci USA* **87**: 2700–2704

Kim JH, Ho SB, Montgomery CK and Kim S (1990) Cell lineage markers in human pancreatic cancer. *Cancer* **66**: 2134–2143

Kliewer SA, Umesono K, Mangelsdorf DJ and Evans RM (1992) Retinoid X receptor interacts with nuclear receptors in retinoic acid, thyroid hormone and vitamin D3 signalling. *Nature* **355**: 446–449

Lee X, Si SP, Tsou HC and Peacocke M (1995) Cellular aging and transformation suppression: a role for retinoic acid receptor β 2. *Exp Cell Res* **218**: 296–304

Leid M, Kastner P and Chambon P (1992*a*) Multiplicity generates diversity in the retinoic acid signaling pathway. *Trends Biochem Sci* **17**: 427–433

Leid M, Kastner P, Lyons R, Naksharti H, Saunders M, Zacherewski T, Chen JY, Staub A, Garnier JM and Chambon P (1992*b*) Purification, cloning and RXR identity of the HeLa cell factor with which RAR or TR heterodimers bind target sequences effectively. *Cell* **68**: 377–395

Lionetto R, Pugliese V, Bruzzi P and Rosso R (1995) No standard treatment is available for advanced pancreatic cancer. *Eur J Cancer* **31A**: 882–887

Longnecker DS and Curphey TJ (1975) Adenocarcinoma of the pancreas in azaserine-treated rats. *Cancer Res* **35**: 2249–2258

Longnecker DS, Memoli V and Pettengill OS (1992) Recent results in animal models of pancreatic carcinoma: histogenesis of tumors. *Yale J Biol Med* **65**: 457–464

Lotan R (1980) Effects of vitamin A and its analogs (retinoids) on normal and neoplastic cells. *Biochim Biophys Acta* **605**: 33–91

Lotan R, Lotan D and Sacks PG (1990) Inhibition of tumor cell growth by retinoids. *Methods Enzymol* **190**: 100–110

Love JM and Gudas LJ (1994) Vitamin A, differentiation and cancer. *Curr Opin Cell Biol* **6**: 825–831

Mangelsdorf DJ and Evans RM (1995) The RXR heterodimers and orphan receptors. *Cell* **83**: 841–850

Mangelsdorf DJ, Borgmeyer U, Heyman RA, Zhou JY, Ong ES, Oro AE, Kakizuka A and Evans RM (1992) Characterization of three RXR genes that mediate the action of 9-cis retinoic acid. *Genes Dev* **6**: 329–344

Mangelsdorf DJ, Umesono K and Evans R (1994) The retinoid receptors. In *The Retinoids: Biology, Chemistry and Medicine*, 2nd edn. Mangelsdorf JE, Evans R (eds), pp. 319–349. Raven Press: New York

Marshall GM, Cheung B, Stacey KP, Camacho ML, Simpson AM, Kwan E, Smith S, Haber M and Norris MD (1995) Increased retinoic acid receptor γ expression suppresses the malignant phenotype and alters the differentiation potential of human neuroblastoma cells. *Oncogene* **11**: 485–491

Moasser MM, DeBlasio A and Dmitrovsky E (1994) Response and resistance to retinoic acid are mediated through the retinoic acid nuclear receptor γ in human teratocarcinomas. *Oncogene* **9**: 833–840

Muindi JR, Frankel SR, Huselton C, DeGrazia F, Garland WA, Young CW and Warrell Jr RP (1992) Clinical pharmacology of oral all-trans retinoic acid in patients with acute promyelocytic leukemia. *Cancer Res* **52**: 2138–2142

Näär AM, Boutin JM, Lipkin SM, Yu VC, Holloway JM, Glass CK and Rosenfeld MG (1991) The orientation and spacing of core DNA-binding motifs dictate selective transcriptional responses to three nuclear receptors. *Cell* **65**: 1267–1279

Pettengill OS, Faris RA, Bell Jr RH, Kuhlmann ET and Longnecker DS (1993) Derivation of ductlike cell lines from a transplantable acinar cell carcinoma of the rat pancreas. *Am J Pathol* **143**: 292–303

Rosewicz S, Lewis LD, Wang XY, Liddle RA and Logsdon CD (1989) Pancreatic digestive enzyme gene expression: effects of CCK and soybean trypsin inhibitor. *Am J Physiol* **256**: G733–738

Rosewicz S, Riecken EO and Wiedenmann B (1992*a*) The amphicrine pancreatic cell line AR42J: a model system for combined studies on exocrine and endocrine secretion. *Clin Invest* **70**: 205–209

Rosewicz S, Vogt D, Harth N, Grund C, Franke WW, Ruppert S, Schweitzer E, Riecken EO and Wiedenmann B (1992*b*) An amphicrine pancreatic cell line: AR42J cells combine exocrine and neuroendocrine properties. *Eur J Cell Biol* **59**: 80–91

Rosewicz S, Detjen K, Kaiser A, Prosenc N, Cervos-Navarro J, Riecken EO and Haller H (1994) Bombesin receptor gene expression in rat pancreatic acinar AR42J cells: transcriptional regulation by glucocorticoids. *Gastroenterology* **107**: 208–218

Rosewicz S, Stier U, Brembeck F, Kaiser A, Papadimitriou CA, Berdel WE, Wiedenmann B and Riecken EO (1995*a*) Retinoids: effects on growth, differentiation, and nuclear receptor expression in human pancreatic carcinoma cell lines. *Gastroenterology* **109**: 1646–1660

Rosewicz S, Riecken EO and Stier U (1995*b*) Transcriptional regulation of carbonic anhydrase II by retinoic acid in the human pancreatic tumor cell line DANG. *FEBS Lett* **368**: 45–48

Satake K, Mukai R, Kato Y, Shim K and Umeyama K (1984) Experimental pancreatic carcinoma as a model of human pancreatic carcinoma. *Clin Oncol* **10**: 27–34

Scarpelli DG, Rao MS and Reddy JK (1984) Studies of pancreatic carcinogenesis in different animal models. *Environ Health Perspect* **56**: 217–227

Smith MA, Parkinson DR, Cheson BD and Friedman MA (1992) Retinoids in cancer therapy. *J Clin Oncol* **10**: 839–864

Tallman MS and Wiernik PH (1992) Retinoids in cancer treatment. *J Clin Pharmacol* **32**: 868–888

Umesono K, Murakami KK, Thompson CC and Evans RM (1991) Direct repeats as selective response elements for the thyroid hormone, retinoic acid, and vitamin D3 receptors. *Cell* **65**: 1255–1266

van der Leede BJ, Folkers GE, van den Brink CE, van der Saag PT and van der Burg B (1995) Retinoic acid receptor α 1 isoform is induced by estradiol and confers retinoic acid sensitivity in human breast cancer cells. *Mol Cell Endocrinol* **109**: 77–86

Xu XC, Stier U, Rosewicz S, El-Naggar AK and Lotan R (1996) Selective suppression of nuclear retinoic acid receptor β gene expression in human pancreatic carcinomas. *Int J Oncol* **8**: 445–451

Zelent A, Mendelsohn C, Kastner P, Krust A, Garnier JM, Ruffenach F, Leroy P and Chambon P (1991) Differentially expressed isoforms of the mouse retinoic acid receptor β are generated by usage of two promoters and alternative splicing. *EMBO J* **10**: 71–78

© Cancer Research Campaign 1998


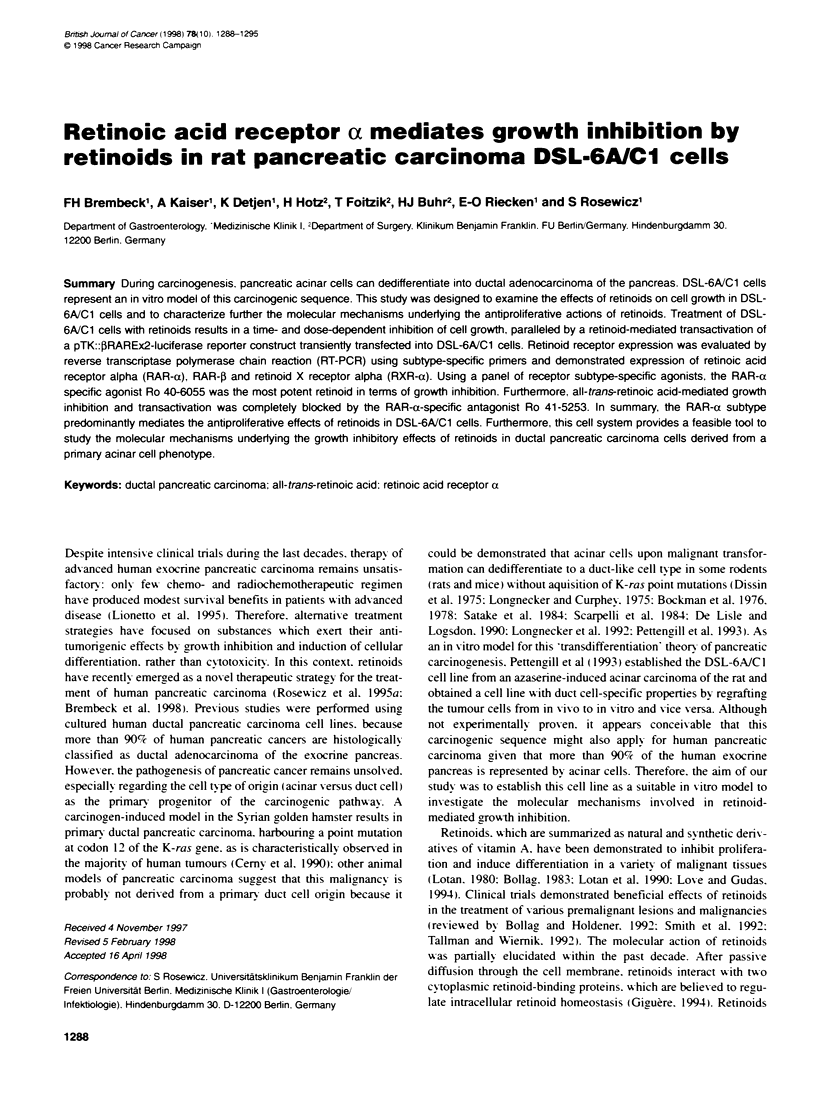

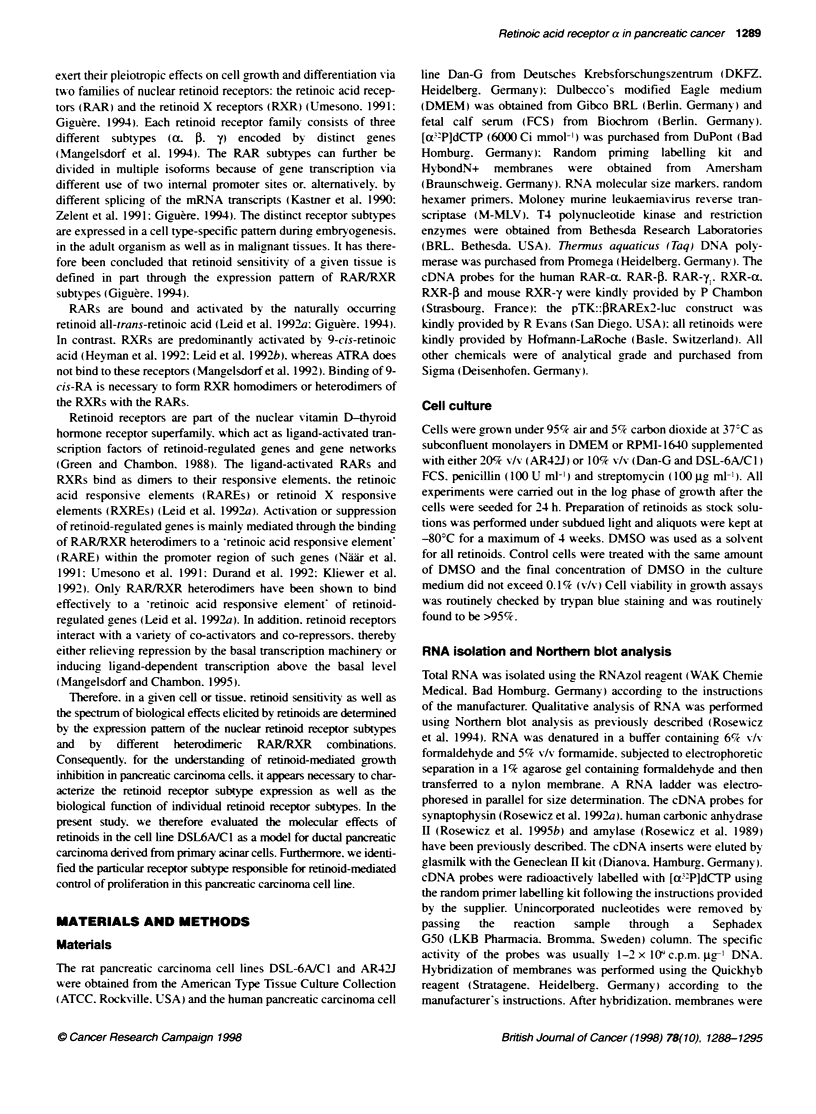

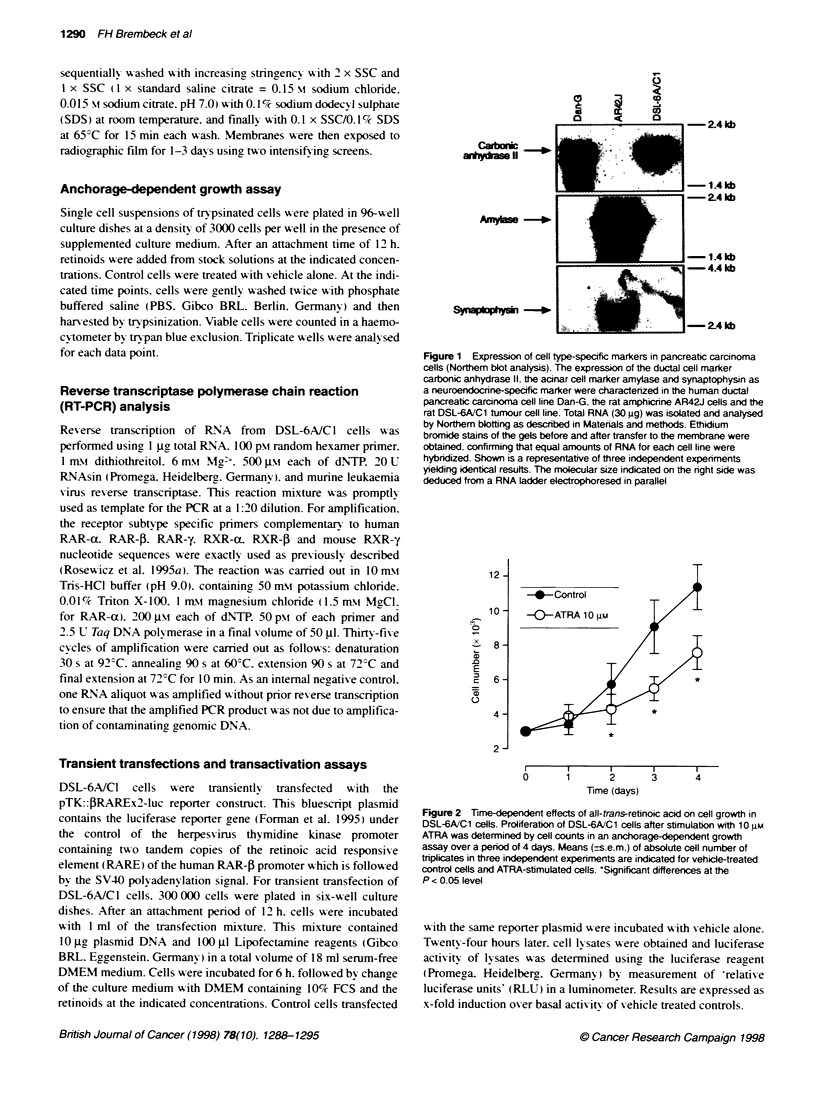

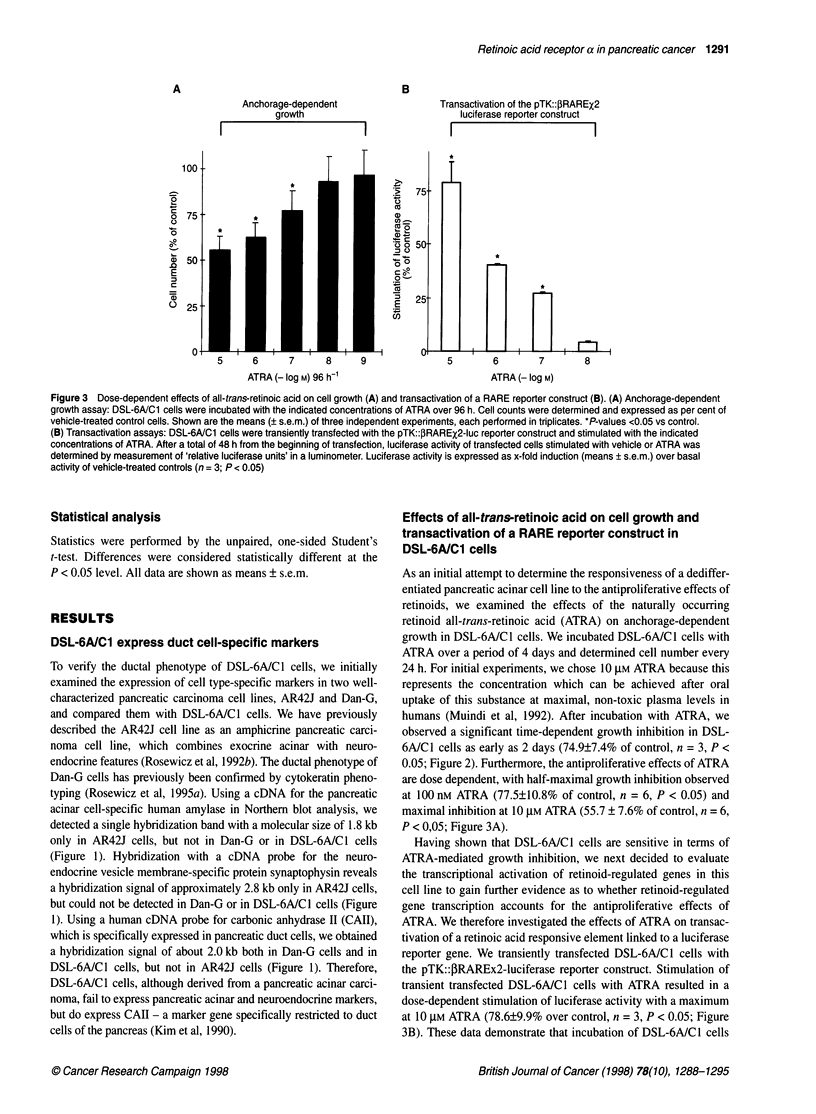

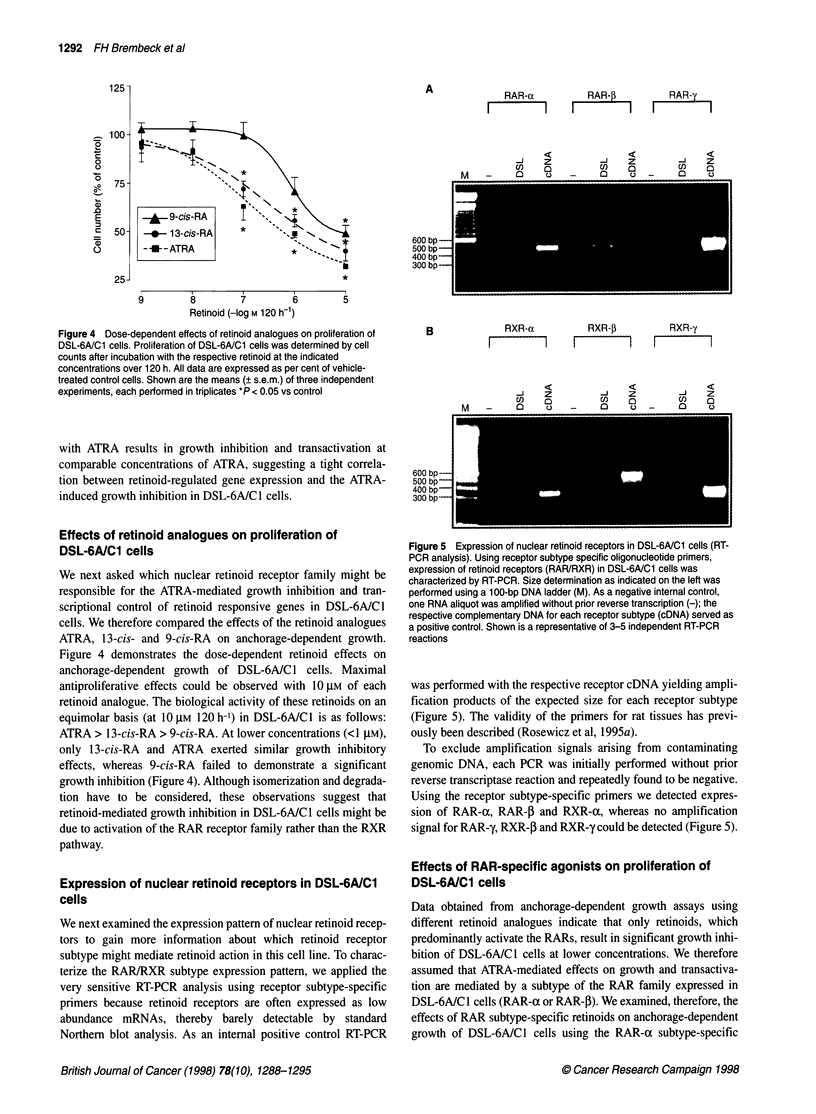

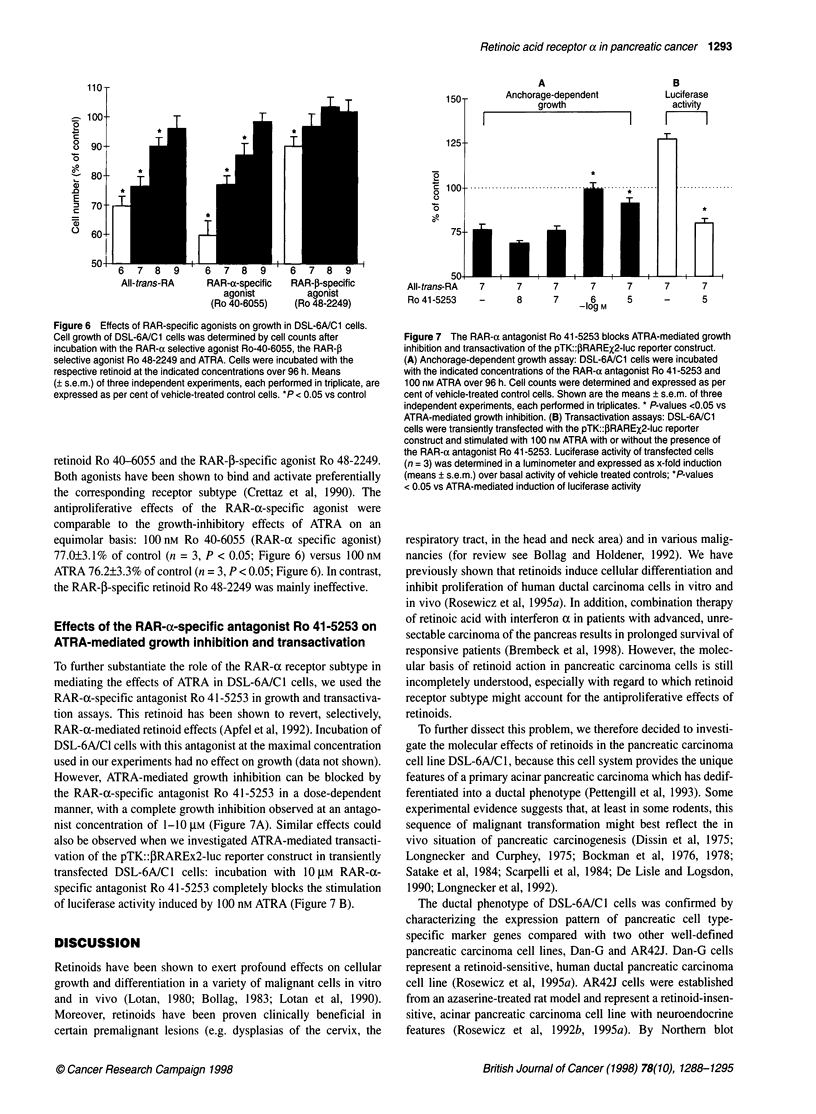

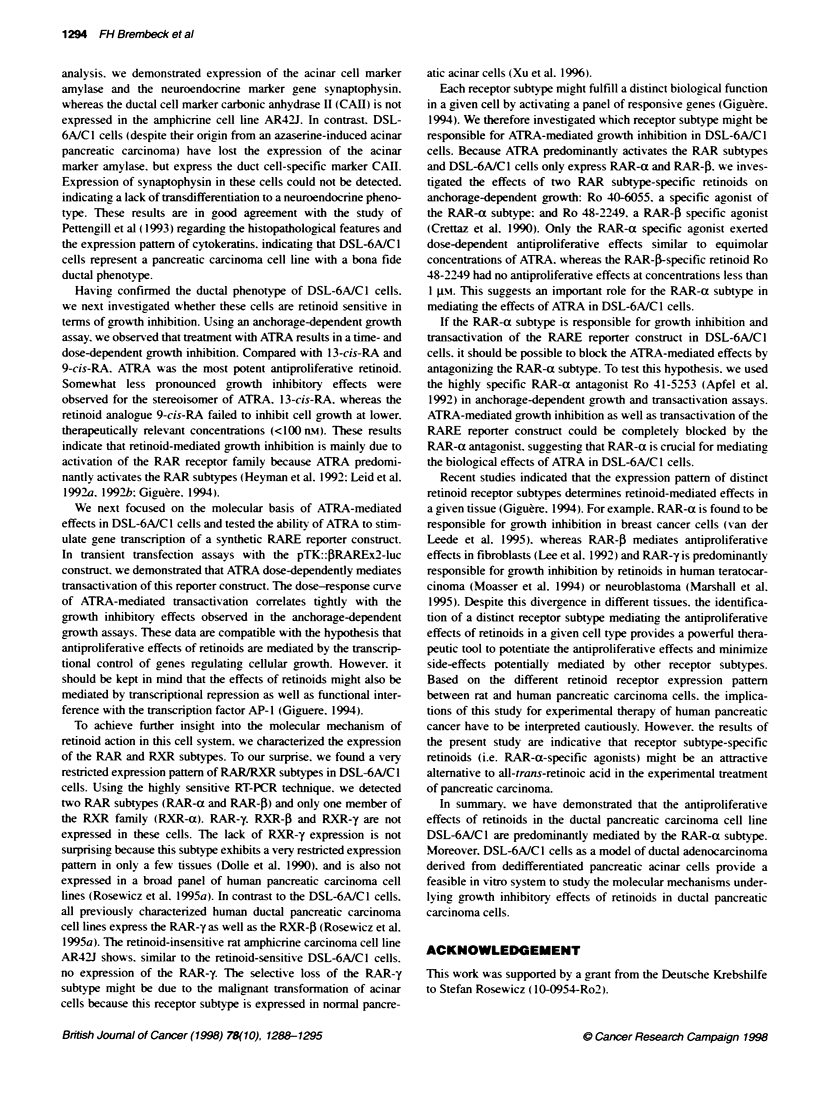

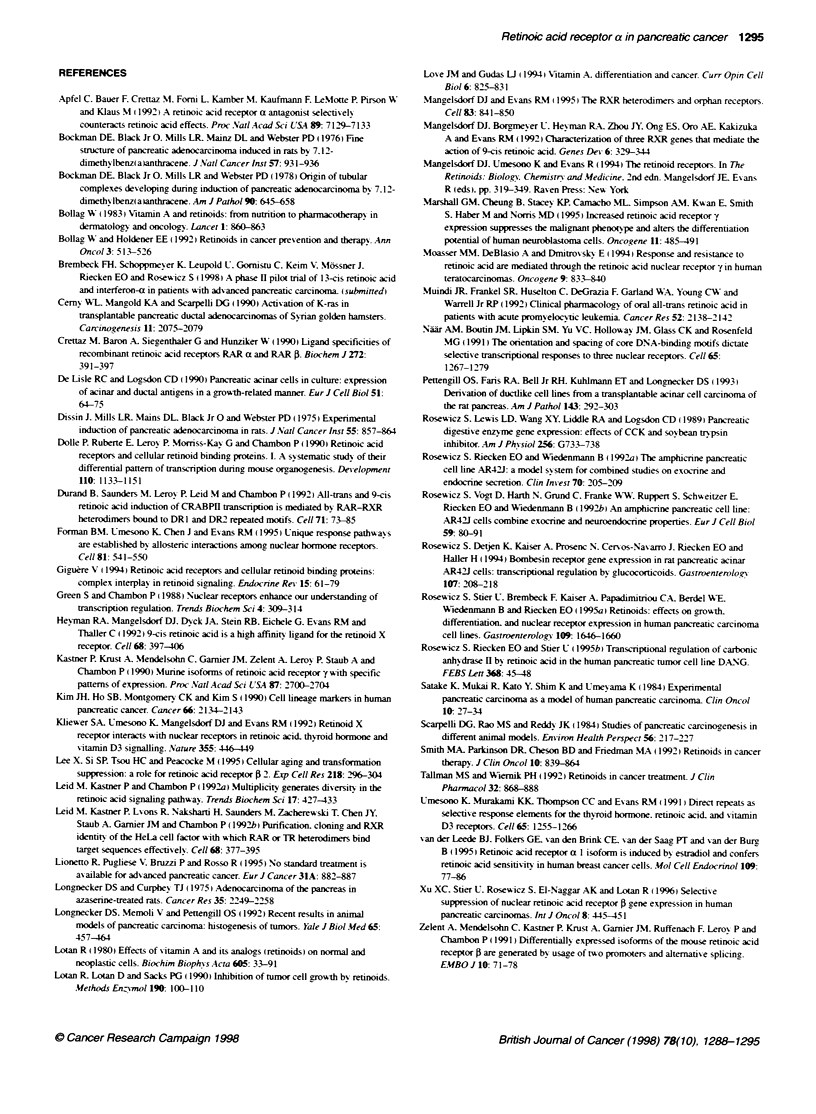


## References

[OCR_00986] Apfel C., Bauer F., Crettaz M., Forni L., Kamber M., Kaufmann F., LeMotte P., Pirson W., Klaus M. (1992). A retinoic acid receptor alpha antagonist selectively counteracts retinoic acid effects.. Proc Natl Acad Sci U S A.

[OCR_00987] Bockman D. E., Black O., Mills L. R., Mainz D. L., Webster P. D. (1976). Fine structure of pancreatic adenocarcinoma induced in rats by 7,12-dimethylbenz(a)anthracene.. J Natl Cancer Inst.

[OCR_00994] Bockman D. E., Black O., Mills L. R., Webster P. D. (1978). Origin of tubular complexes developing during induction of pancreatic adenocarcinoma by 7,12-dimethylbenz(a)anthracene.. Am J Pathol.

[OCR_01003] Bollag W., Holdener E. E. (1992). Retinoids in cancer prevention and therapy.. Ann Oncol.

[OCR_01011] Cerny W. L., Mangold K. A., Scarpelli D. G. (1990). Activation of K-ras in transplantable pancreatic ductal adenocarcinomas of Syrian golden hamsters.. Carcinogenesis.

[OCR_01016] Crettaz M., Baron A., Siegenthaler G., Hunziker W. (1990). Ligand specificities of recombinant retinoic acid receptors RAR alpha and RAR beta.. Biochem J.

[OCR_01021] De Lisle R. C., Logsdon C. D. (1990). Pancreatic acinar cells in culture: expression of acinar and ductal antigens in a growth-related manner.. Eur J Cell Biol.

[OCR_01026] Dissin J., Mills L. R., Mains D. L., Black O., Webster P. D. (1975). Experimental induction of pancreatic adenocarcinoma in rats.. J Natl Cancer Inst.

[OCR_01029] Dollé P., Ruberte E., Leroy P., Morriss-Kay G., Chambon P. (1990). Retinoic acid receptors and cellular retinoid binding proteins. I. A systematic study of their differential pattern of transcription during mouse organogenesis.. Development.

[OCR_01034] Durand B., Saunders M., Leroy P., Leid M., Chambon P. (1992). All-trans and 9-cis retinoic acid induction of CRABPII transcription is mediated by RAR-RXR heterodimers bound to DR1 and DR2 repeated motifs.. Cell.

[OCR_01039] Forman B. M., Umesono K., Chen J., Evans R. M. (1995). Unique response pathways are established by allosteric interactions among nuclear hormone receptors.. Cell.

[OCR_01044] Giguère V. (1994). Retinoic acid receptors and cellular retinoid binding proteins: complex interplay in retinoid signaling.. Endocr Rev.

[OCR_01048] Green S., Chambon P. (1988). Nuclear receptors enhance our understanding of transcription regulation.. Trends Genet.

[OCR_01059] Kastner P., Krust A., Mendelsohn C., Garnier J. M., Zelent A., Leroy P., Staub A., Chambon P. (1990). Murine isoforms of retinoic acid receptor gamma with specific patterns of expression.. Proc Natl Acad Sci U S A.

[OCR_01062] Kim J. H., Ho S. B., Montgomery C. K., Kim Y. S. (1990). Cell lineage markers in human pancreatic cancer.. Cancer.

[OCR_01068] Kliewer S. A., Umesono K., Mangelsdorf D. J., Evans R. M. (1992). Retinoid X receptor interacts with nuclear receptors in retinoic acid, thyroid hormone and vitamin D3 signalling.. Nature.

[OCR_01073] Lee X., Si S. P., Tsou H. C., Peacocke M. (1995). Cellular aging and transformation suppression: a role for retinoic acid receptor beta 2.. Exp Cell Res.

[OCR_01076] Leid M., Kastner P., Chambon P. (1992). Multiplicity generates diversity in the retinoic acid signalling pathways.. Trends Biochem Sci.

[OCR_01080] Leid M., Kastner P., Lyons R., Nakshatri H., Saunders M., Zacharewski T., Chen J. Y., Staub A., Garnier J. M., Mader S. (1992). Purification, cloning, and RXR identity of the HeLa cell factor with which RAR or TR heterodimerizes to bind target sequences efficiently.. Cell.

[OCR_01084] Lionetto R., Pugliese V., Bruzzi P., Rosso R. (1995). No standard treatment is available for advanced pancreatic cancer.. Eur J Cancer.

[OCR_01089] Longnecker D. S., Curphey T. J. (1975). Adenocarcinoma of the pancreas in azaserine-treated rats.. Cancer Res.

[OCR_01093] Longnecker D. S., Memoli V., Pettengill O. S. (1992). Recent results in animal models of pancreatic carcinoma: histogenesis of tumors.. Yale J Biol Med.

[OCR_01096] Lotan R. (1980). Effects of vitamin A and its analogs (retinoids) on normal and neoplastic cells.. Biochim Biophys Acta.

[OCR_01102] Lotan R., Lotan D., Sacks P. G. (1990). Inhibition of tumor cell growth by retinoids.. Methods Enzymol.

[OCR_01115] Mangelsdorf D. J., Borgmeyer U., Heyman R. A., Zhou J. Y., Ong E. S., Oro A. E., Kakizuka A., Evans R. M. (1992). Characterization of three RXR genes that mediate the action of 9-cis retinoic acid.. Genes Dev.

[OCR_01108] Mangelsdorf D. J., Evans R. M. (1995). The RXR heterodimers and orphan receptors.. Cell.

[OCR_01122] Marshall G. M., Cheung B., Stacey K. P., Camacho M. L., Simpson A. M., Kwan E., Smith S., Haber M., Norris M. D. (1995). Increased retinoic acid receptor gamma expression suppresses the malignant phenotype and alters the differentiation potential of human neuroblastoma cells.. Oncogene.

[OCR_01129] Moasser M. M., DeBlasio A., Dmitrovsky E. (1994). Response and resistance to retinoic acid are mediated through the retinoic acid nuclear receptor gamma in human teratocarcinomas.. Oncogene.

[OCR_01136] Muindi J. R., Frankel S. R., Huselton C., DeGrazia F., Garland W. A., Young C. W., Warrell R. P. (1992). Clinical pharmacology of oral all-trans retinoic acid in patients with acute promyelocytic leukemia.. Cancer Res.

[OCR_01139] När A. M., Boutin J. M., Lipkin S. M., Yu V. C., Holloway J. M., Glass C. K., Rosenfeld M. G. (1991). The orientation and spacing of core DNA-binding motifs dictate selective transcriptional responses to three nuclear receptors.. Cell.

[OCR_01147] Pettengill O. S., Faris R. A., Bell R. H., Kuhlmann E. T., Longnecker D. S. (1993). Derivation of ductlike cell lines from a transplantable acinar cell carcinoma of the rat pancreas.. Am J Pathol.

[OCR_01169] Rosewicz S., Detjen K., Kaiser A., Prosenc N., Cervos-Navarro J., Riecken E. O., Haller H. (1994). Bombesin receptor gene expression in rat pancreatic acinar AR42J cells: transcriptional regulation by glucocorticoids.. Gastroenterology.

[OCR_01150] Rosewicz S., Lewis L. D., Wang X. Y., Liddle R. A., Logsdon C. D. (1989). Pancreatic digestive enzyme gene expression: effects of CCK and soybean trypsin inhibitor.. Am J Physiol.

[OCR_01180] Rosewicz S., Riecken E. O., Stier U. (1995). Transcriptional regulation of carbonic anhydrase II by retinoic acid in the human pancreatic tumor cell line DANG.. FEBS Lett.

[OCR_01155] Rosewicz S., Riecken E. O., Wiedenmann B. (1992). The amphicrine pancreatic cell line AR42J: a model system for combined studies on exocrine and endocrine secretion.. Clin Investig.

[OCR_01177] Rosewicz S., Stier U., Brembeck F., Kaiser A., Papadimitriou C. A., Berdel W. E., Wiedenmann B., Riecken E. O. (1995). Retinoids: effects on growth, differentiation, and nuclear receptor expression in human pancreatic carcinoma cell lines.. Gastroenterology.

[OCR_01162] Rosewicz S., Vogt D., Harth N., Grund C., Franke W. W., Ruppert S., Schweitzer E., Riecken E. O., Wiedenmann B. (1992). An amphicrine pancreatic cell line: AR42J cells combine exocrine and neuroendocrine properties.. Eur J Cell Biol.

[OCR_01187] Satake K., Mukai R., Kato Y., Shim K., Umeyama K. (1984). Experimental pancreatic carcinoma as a model of human pancreatic carcinoma.. Clin Oncol.

[OCR_01192] Scarpelli D. G., Rao M. S., Reddy J. K. (1984). Studies of pancreatic carcinogenesis in different animal models.. Environ Health Perspect.

[OCR_01196] Smith M. A., Parkinson D. R., Cheson B. D., Friedman M. A. (1992). Retinoids in cancer therapy.. J Clin Oncol.

[OCR_01200] Tallman M. S., Wiernik P. H. (1992). Retinoids in cancer treatment.. J Clin Pharmacol.

[OCR_01202] Umesono K., Murakami K. K., Thompson C. C., Evans R. M. (1991). Direct repeats as selective response elements for the thyroid hormone, retinoic acid, and vitamin D3 receptors.. Cell.

[OCR_01220] Zelent A., Mendelsohn C., Kastner P., Krust A., Garnier J. M., Ruffenach F., Leroy P., Chambon P. (1991). Differentially expressed isoforms of the mouse retinoic acid receptor beta generated by usage of two promoters and alternative splicing.. EMBO J.

[OCR_01207] van der Leede B. J., Folkers G. E., van den Brink C. E., van der Saag P. T., van der Burg B. (1995). Retinoic acid receptor alpha 1 isoform is induced by estradiol and confers retinoic acid sensitivity in human breast cancer cells.. Mol Cell Endocrinol.

